# Evaluation of a fully automated assay for the measurement of plasma pTau217 and a composite score integrating the ratio Aβ1-42/1-40 as biomarkers of Alzheimer’s disease

**DOI:** 10.1007/s00406-025-02123-8

**Published:** 2025-09-27

**Authors:** Barbara Morgado, Hans-Wolfgang Klafki, Chris Bauer, Annik Steiert, Katharina Waniek, Ingolf Lachmann, Dirk Osterloh, Hermann Esselmann, Niels Hansen, Johannes Schuchhardt, Jens Wiltfang

**Affiliations:** 1 Department of Psychiatry and Psychotherapy, University Medical Center Goettingen (UMG), Georg-August University, Von-Siebold Strasse 5, 37075 Goettingen, Germany; 2 MicroDiscovery GmbH, Marienburger Strasse 1, 10405 Berlin, Germany; 3 Roboscreen GmbH, Hohmannstrasse 7, 04129 Leipzig, Germany; 4 German Center for Neurodegenerative Diseases (DZNE), 37075 Goettingen, Germany; 5 Neurosciences and Signaling Group, Institute of Biomedicine (iBiMED), Department of Medical Sciences, University of Aveiro, 3810-193 Aveiro, Portugal

**Keywords:** Alzheimer’s disease, Blood-based biomarker, pTau217, pTau181, Aβ1-40/Aβ1-42*pTau217 composite marker, Pre-analytical immunoprecipitation

## Abstract

**Background:**

Plasma phosphorylated Tau217 (pTau217) represents a sensitive blood-based biomarker for Alzheimer’s disease (AD). This study investigated the performance of plasma pTau217 alone and as a composite score (Aβ1-40/Aβ1-42*pTau217) for detecting low Aβ1-42/1-40 in cerebrospinal fluid (CSF) as a surrogate marker of brain β-amyloid pathological changes.

**Methods:**

We analysed plasma samples from 82 pre-selected participants who were dichotomized according to their CSF Aβ42/40 ratio after data-driven cutoff determination by Gaussian mixture modelling. The study cohort included patients in very early disease stages with mild cognitive impairment (MCI) due to AD and mild dementia due to AD in the Aβ-positive group and MCI due to other causes and mild dementia due to other causes in the Aβ-negative group. Concentrations of plasma pTau181 and pTau217 were determined on the fully automated LUMIPULSE platform. Additionally, plasma Aβ1-42/1-40 and pTau217 were assessed after consecutive Aβ- and Tau-immunoprecipitations (IPs). Corrections for age and sex effects in primary variables were done using a multivariate linear model on a logarithmic scale. Results are reported using both adjusted and unadjusted values.

**Results:**

After adjustment for age and sex, median plasma pTau217 was increased by 203% in participants with low CSF Aβ1-42/1-40 (i.e. Aβ-positive), while pTau181 was increased by only 61%. PTau217 showed a larger area under the receiver operating characteristics curve (AUC) of 0.88 in identifying Aβ-positive individuals compared to plasma pTau181 (AUC: 0.78). Plasma Aβ1-42/1-40 ratio in Aβ-IP eluates (adjusted for age and sex) was decreased by only 10%, and the standardized effect size (Cohen´s d) was smaller than that of pTau217. Tau-IP did not improve the performance of the pTau217 assay (p = 0.568, DeLong test). However, the Aβ1-40/Aβ1-42*pTau217 ratio following immunoprecipitations appeared to outperform the single blood biomarkers.

**Conclusion:**

PTau217 as measured with the fully automated Lumipulse G pTau217 Plasma < RUO > assay demonstrated high accuracy for detecting low CSF Aβ1-42/1-40 and appears to represent a particularly attractive biomarker for future implementation in clinical practice. Furthermore, the composite score Aβ1-40/Aβ1-42*pTau217 showed promise for enhancing the blood-based early and differential diagnosis of AD.

**Supplementary Information:**

The online version contains supplementary material available at 10.1007/s00406-025-02123-8.

## Introduction

Blood biomarkers that can detect the key pathological hallmarks of Alzheimer’s disease (AD) are currently emerging as early diagnostic tools. The accurate and robust measurements of AD biomarkers in blood through reliable high-throughput platforms have the potential for a wide range of applications [1] including screening, early diagnosis, tracking progression, and monitoring the efficacy of the recent developments regarding disease-modifying therapies [2–6].

Recently, the Alzheimer’s Association extended the diagnostic criteria of AD based on its biological markers rather than clinical symptoms from research context to routine diagnostic practice [7]. AD is understood as a disease continuum, based on a temporal dynamic biomarker model suggesting a progressive increase of pathological burden over time ultimately leading to the manifestation of clinical symptoms [8]. Aβ deposition is the earliest pathological change detected which makes it a very relevant biomarker in early disease stages [9, 10]. Well-established biomarkers reflecting the brain Aβ proteinopathy include a low Aβ42/40 ratio in cerebrospinal fluid (CSF) and increased signals on amyloid-positron emission tomography (amyloid-PET).

Phosphorylated forms of Tau (pTau), such as pTau181 and pTau217, have been proposed as other neurochemical biomarkers of very early AD pathology and are strongly associated with Aβ pathological changes [11, 12]. These analytes become abnormal around the same time as amyloid PET and precede detectable Tau pathology, as measured by Tau PET [13–16]. Thus, due to the onset time, elevated soluble pTau has been proposed as a surrogate biomarker of amyloid pathology [17]. Numerous studies have shown diagnostic superiority of pTau217 over pTau181, with a greater fold change in symptomatic AD patients compared to controls and better predictive ability regarding cognitive decline [18–20]. In plasma, pTau217 is already elevated in cognitively unimpaired individuals with evidence of preclinical brain Aβ neuropathology [2] as well as in cognitively impaired individuals with biomarker evidence of amyloid deposition [21] (i.e. AD-dementia or mild cognitive impairment (MCI) due to AD) and can accurately differentiate dementia due to AD from other dementias [22]. Moreover, changes in the levels of pTau217 in plasma seem to be detectable approximately at the same time as in CSF [23] and with similar performance in identifying AD pathological changes [22].

As the interest in implementing blood-based biomarkers in research and clinical care increases, novel technologies are being developed and becoming more widely available. In the present study, we employed a novel Lumipulse G pTau 217 Plasma assay for Research Use Only (RUO) (Fujirebio) to measure plasma pTau217 in a preselected clinical sample set to detect CSF biomarker evidence of AD pathological changes. Its performance was compared to a commercially available plasma pTau181 immunoassay on the same platform. Finally, we tested the impact of pre-analytical magnetic bead Tau immunoprecipitation (IP) on the performance of the plasma pTau217 biomarker assay.

## Materials and methods

### Study participants and study approval

The archived plasma samples assessed in this study were obtained from the local biobank at the Department of Psychiatry and Psychotherapy at the University Medical Center Goettingen/University of Goettingen. The pseudonymized collection of biological samples and clinical data in the biobank followed the revised Declaration of Helsinki, Good Clinical Practice guidelines and the standard operating procedures (SOPs) of the German Center for Neurodegenerative Diseases (DZNE) [24]. Their use in biomarker studies was approved by the ethics committee of the University Goettingen (9/2/16), and individual informed consent was obtained from all individuals or their legal representatives. In detail, before inclusion in the study and removal of biomaterial, all study participants received detailed information and an information leaflet from the study physicians at the Department of Psychiatry and Psychotherapy, University Medical Center Göttingen, before signing a written informed consent form. All study participants had MCI or mild dementia, so that they were able to give informed consent. If the ability to consent was not given, a separate informed consent form was also provided for the study participant's legal representative, and the legal representative signed the informed consent form for the study. The ethics vote has the AZ 9/2/16.

### Recruitment strategy and clinical and neuropsychological assessments

The initial selection of plasma samples for the study was based on a biomarker-supported clinical diagnosis comprising clinical and neuropscychological observations, CSF biomarker data supporting AD or non-AD (Aβ42/40 ratio, pTau181 and total Tau measured routinely in the Clinical Neurochemistry and Neurochemical Dementia Diagnostics Laboratory at the Department of Psychiatry and Psychotherapy, University of Erlangen-Nuremberg, Germany), psychometric data (n = 82) and, when available, imaging biomarker data (MRI, n = 29; Amyloid-PET, n = 10). The neuropsychological examination comprised various tests in most participants, including CERAD-NAB Plus (Consortium to Establish a Registry for Alzheimer’s Disease Neuropsychological Assessment Battery plus), parts of the WMS-IV (Wechsler Memory Scale – 4th edition) on visual reproduction and logical memory, WAIS-IV (Wechsler Adult Intelligance Scale – 4th edition) for forward and backward digit span, block structure and encoding, and RWT (Regensburg word fluency test, which measures semantic and formal lexical fluency. As one of the 10 clinical centers of the DZNE (https://www.dzne.de/en/research/studies/clinical-studies/delcode, accessed on 31.03.2025), our recruitment strategy and diagnostic criteria are based on the DZNE guidelines and are specified by Jessen et al. [24] as part of the DELCODE study (Longitudinal Cognitive Impairment and Dementia Study) for early AD. Specifically, patients of predominantly Caucasian ethnicity were recruited by presentation from their own initiatives, general practitioners, specialists and non-university clinics, primarily in the south-eastern Lower Saxony region from august/ 2016 to august/ 2022. Amnestic mild cognitive impairment was diagnosed by a cognitive performance of less than -1.5 times the standard deviation from the delayed recall trial of the CERAD episodic memory testing procedure using word-lists. Cognitive performance was adjusted for age, gender and education. The research criteria [25] for MCI were thus fulfilled. Early dementia was diagnosed if the MMSE score was ≥ 20. The clinical diagnosis of early symptomatic AD and disease controls were validated by the CSF biomarkers described above. In line with the above recruitment strategy, our selected study sample included 41 patients with low CSF Aβ42/40 and a clinical phenotype of Alzheimer's disease between MCI (n = 24) and mild dementia (n = 17) according to the criteria recommended by the international working group [26]) and 41 control subjects with cognitive impairment between MCI (n = 22) and mild dementia (n = 19) and normal CSF Aβ42/40. Thus, the study cohort included only MCI due to AD and mild dementia due to AD in the amyloid-positive group, and MCI due to other causes and mild dementia due to other causes in the amyloid-negative group.

### Blood-collection procedure

The blood biomaterial was processed and stored according to the standard protocol of the biomaterial bank at the Department of Psychiatry and Psychotherapy at the University Medical Center Göttingen. A fasting protocol was not included in the blood collection procedures. The blood samples were taken and brought to the laboratory within half an hour. There, they were processed in the laboratory immediately upon receipt. EDTA plasma was centrifuged at 2000 × g for 10 min at 20 °C (Hereaus Multifuge X3R). The plasma was then stored in 500 µL aliquots. The biomaterial was stored at -80 °C in a freezer in conjunction with the emergency power system of the University Medical Center Göttingen and a CO_2_ emergency cooling system.

### ApoE genotyping

ApoE genotyping was performed using a modified quantitative real-time PCR protocol as previously described [27]. DNA was prepared from whole blood with the DNeasy Blood & Tissue kit (Qiagen) and analysed on a CFX Connect Real-Time PCR system using the iTaq™ Universal SYBR Green Supermix (Bio-Rad). All measurements were performed in duplicates for all primer combinations including negative controls.

### Preparation of functionalized magnetic beads

Functionalized magnetic beads for Aβ-immunoprecipitation (Aβ-IP) were produced by covalently coupling the monoclonal antibody (mAb) 1E8 (nanoTools, Teningen, Germany) with Dynabeads M-280 Sheep anti-Mouse IgG (Invitrogen/ThermoFisher Scientific, Waltham, MA, USA) according to the manufacturer’s instructions and as described in detail previously [28].

For Tau-IP, functionalized magnetic beads were produced by coupling the mAbs 2B8 and 7E5 (Roboscreen, Leipzig, Germany) with Dynabeads M-270 Epoxy (Invitrogen/ ThermoFisher Scientific) according to manufacturer’s protocol and as previously described [29]. mAb 2B8 binds to brain derived Tau against the amino acids 121–128 (HVTQARMV) while mAb 7E5 recognizes the amino acids 156–165 (RGAAPPGQKGQA) of 2N4R Tau.

### Aβ and Tau-immunoprecipitations

The semi-automated pre-analytical Aβ-IP from EDTA-blood plasma was performed on a CyBio FeliX liquid handling instrument (Analytik Jena, Jena, Germany) following the protocol previously described [29]. In summary, aliquots of EDTA-blood plasma stored at − 80 °C in Matrix 0.5 mL tubes (Thermo Scientific) were thawed at room temperature (RT), mixed vigorously for 5–10 s and centrifuged for 10 min at 10,000 × *g* at RT in a fixed angle rotor for removal of insoluble material. 200 μL of the plasma supernatant was mixed in a 96-deepwell sample plate (Sarstedt, Nümbrecht, Germany) with 200 µL H_2_O, 100 µL of 5 × IP-buffer concentrate (250 mM HEPES/NaOH, pH 7.4, 750 mM NaCl, 2.5% Igepal CA630, 1.25% sodium deoxycholate, 0.25% SDS and Complete Mini Protease inhibitor cocktail (Roche)) and 25 µL of functionalized 1E8 magnetic beads (see above). The IP reaction was carried out on an Eppendorf ThermoMixer C (Eppendorf, Hamburg, Germany) overnight at 4 °C with continuous agitation at 1,000 rpm. Then, the magnetic beads were immobilized using an ALPAQUA MAGNUM FLX Universal Magnet (Beverly, MA, USA) adapter on the FeliX instrument, and the supernatants (unbound material) were collected and stored at -80 °C for the subsequent Tau-IP. The beads were washed 3 × for 5 min with 1 mL of PBS/0.1% BSA and 1 × for 3 min with 1 mL of 10 mM Tris/HCl, pH 7.5. The bound Aβ peptides were eluted from the 1E8 magnetic beads by adding 2 × 25 μL of PBS containing 0.05% Tween-20 (PBS-T) and heating the 96-deepwell round bottom plate (Sarstedt, Germany) without a lid on a BioShake 3000-T elm (QInstruments, Germany) for 5 min at 99 °C and 1100 rpm. Per sample, approximately 35 μL of bead-free Aβ eluate was obtained and diluted with 265 µL of Specimen Diluent (Fujirebio). 300 µL of diluted IP eluate was manually transferred to 2 mL screw cap micro tubes (Sarstedt, Germany) and stored at -80 °C until the Aβ measurements on the LUMIPULSE G System.

The remaining unbound fractions (collected after the Aβ-IP and stored at -80 °C as described above) were thawed at RT and mixed with 25 µL Tau magnetic beads on the CyBio FeliX liquid handling robot (see above). Tau IP was carried out on the FeliX instrument for 1 h at RT with periodical mixing by up and down pipetting. After the incubation, the beads were collected on the ALPAQUA MAGNUM FLX Universal Magnet, and the supernatants were discarded. The beads were washed 3 × for 5 min with 1 mL of PBS/0.1% BSA and 1 × for 3 min with 1 mL of PBS-T. Finally, Tau proteins were eluted in 2 × 25 µL of PBS-T by heating the 96-deepwell round bottom plate (Sarstedt, Germany) without a lid for 5 min at 99 °C and 1100 rpm in a BioShake 3000-T elm (QInstruments, Germany). To the remaining volume of approximately 38 µL of Tau-IP eluate 220 µL of Lumipulse G Specimen Diluent was added per sample. The diluted IP eluates were transferred to 2 mL screw cap micro tubes (Sarstedt, Germany) and stored at − 80 °C until the pTau217 measurements on the LUMIPULSE G System (Fujirebio).

### Biomarker measurements

For providing a homogeneous CSF biomarker data set for the statistical analyses in this study (and independently of the routine biomarker measurements for clinical diagnosis mentioned above), the CSF concentrations of Aβ1-42, Aβ1-40, total Tau and pTau181 were quantified in single measurements using the CSF Lumipulse G β-Amyloid 1–42, β-Amyloid 1–40, Total Tau and pTau 181 < CE > assays on the LUMIPULSE G automated platform (Fujirebio) and following the manufacturer’s instructions. CSF pTau217 was assessed using a prototype version of the Lumipulse G pTau 217 CSF assay.

For the direct measurements of EDTA-plasma, the concentrations of pTau181 and pTau217 were assessed from centrifuged plasma on the LUMIPULSE G 1200 System at Fujirebio in Gent (Belgium) using the commercially available Lumipulse G pTau 181 Plasma (RUO, Cat# 81,288/81289/81297) and Lumipulse G pTau 217 Plasma (RUO, Cat# 81,472/81471/81473) assays. The plasma pTau217 assay was recently developed and employs magnetic particles coupled to the RD85 mAb, proprietary to Fujirebio, in combination with the detection antibodies against the mid-domain of Tau (HT7 and BT2) labelled with alkaline phosphatase (ALP). The reported limit of detection (LoD) for this novel assay was below 0.020 pg/mL, and the lower limit of quantification (LoQ) was 0.037 pg/mL at a 20% CV level [30].

The immunoprecipitation-immunoassay approach allowed the analysis of pTau217 and Aβ1-42/1-40 biomarkers from the same plasma sample. The levels of pTau217 obtained after pre-analytical Tau-IP were assessed using the aforementioned Lumipulse G pTau217 plasma assay. The measurements of Aβ1–40 and Aβ1–42 peptides on the previously diluted Aβ IP-eluates were performed using the commercial Lumipulse G β-Amyloid 1-40 Plasma and Lumipulse G β-Amyloid 1-42 Plasma assays (RUO, Cat# 81,298/81303/81300 and #81,301/81299/81300, respectively).

### Statistical analysis and classification

The study participants were classified into the groups amyloid-positive and amyloid negative with reference to the Aβ1-42/1-40 ratio in CSF using a cutpoint that was derived from data driven Gaussian mixture modeling. This unbiased approach for determining biomarker cutoffs was introduced in 2010 by De Meyer et al. [31], and has been used in a number of more recent biomarker studies [32–34]. To define amyloid-positivity, two normal distributions were fitted into the distribution of CSF Aβ1-42/1-40 ratios (measured on the Lumipulse platform) with the R package “mix‑tools” (version 1.2.0), and the intersection between the two fitted curves was chosen as the cutoff.

All analyses were performed using R version 4.2.3. Baseline statistics are reported as mean and standard deviation (SD). Scatterplots are shown on logarithmic scale and correlation coefficients are calculated using Spearman rank correlation. For fitting regression lines, we used a Deming regression (R package MethComp version 1.22.2), since both variables were measured experimentally. For the mixture model, we used an EM algorithm for mixtures of normal distributions without further constraints on mean or SD (R package mixtools—version 1.2.0). The biomarker levels in the diagnostic groups Aβ-positive and Aβ-negative were compared using Mann–Whitney tests for numerical variables and Fisher’s exact test for categorical variables. In case of non-normal distribution data, differences between the groups were tested with Wilcoxon test. Corrections for age and sex effects were performed individually for each primary variable using a multivariate linear model, in which the variable was log-transformed, age was included on its original scale, and gender was treated as a categorical variable. Age and sex adjusted values for derived variables (such as the Aβ42/40 ratio in IP-eluates from plasma or AT-Term) were calculated based on the corrected primary variables. Results are reported using both adjusted and unadjusted values.

For assessing the magnitude of the effect size, the relative ${\text{Median difference }}\left( \% \right) = 100*\frac{{{\mathrm{median}} \left( {A\beta + } \right) - {\mathrm{median}} \left( {A\beta - } \right)}}{{{\mathrm{median}}\left( {A\beta - } \right)}}$ and the Cohen’s d (standardised effect size), calculated with R package “effsize” (version 0.8.1), were used. Since we observed a log-normal distribution of relevant variables the Cohen’s d was calculated after logarithmic transformation (log2) in order to assure a correct interpretability of the resulting values.

For testing the significance of the observed difference in effect sizes, we applied a 0.632 bootstrapping (re-sampling of patients with replacement including refinement of the estimator as proposed by Efron in 1983) [35]. We applied 1000 replications of the bootstrapping and calculated the difference in effect sizes (e.g., 0.632 × median difference of the resampling + 0.368 × median difference of data without resampling). The differences between the resulting effect sizes were normally distributed (Shapiro p-value: 0.85). Bootstrapping p-values were calculated by using a normal distribution after normalization of the standard deviation.

The ability of biomarkers to detect amyloid positivity was evaluated using ROC-AUC (area under the receiver operating characteristics curve) analysis. 95% confidence intervals of AUC estimates were calculated and AUC values were compared using the DeLong test. Classification performance was assessed at the Youden point of the ROC curve.

To investigate the combined effect of multiple variables, a multivariate classification of Aβ-positive versus Aβ-negative cases based on biomarker measurements in IP-eluates was done. We constructed a series of logistic regression models with progressively increasing numbers of predictors. For example, we evaluated models including individual biomarkers, combinations such as a biomarker plus ApoE4 allele frequency, and extended models that also incorporated age and sex.

## Results

### CSF AD biomarker measurements on the LUMIPULSE platform and dichotomization of the study sample

**Fig. 1:**
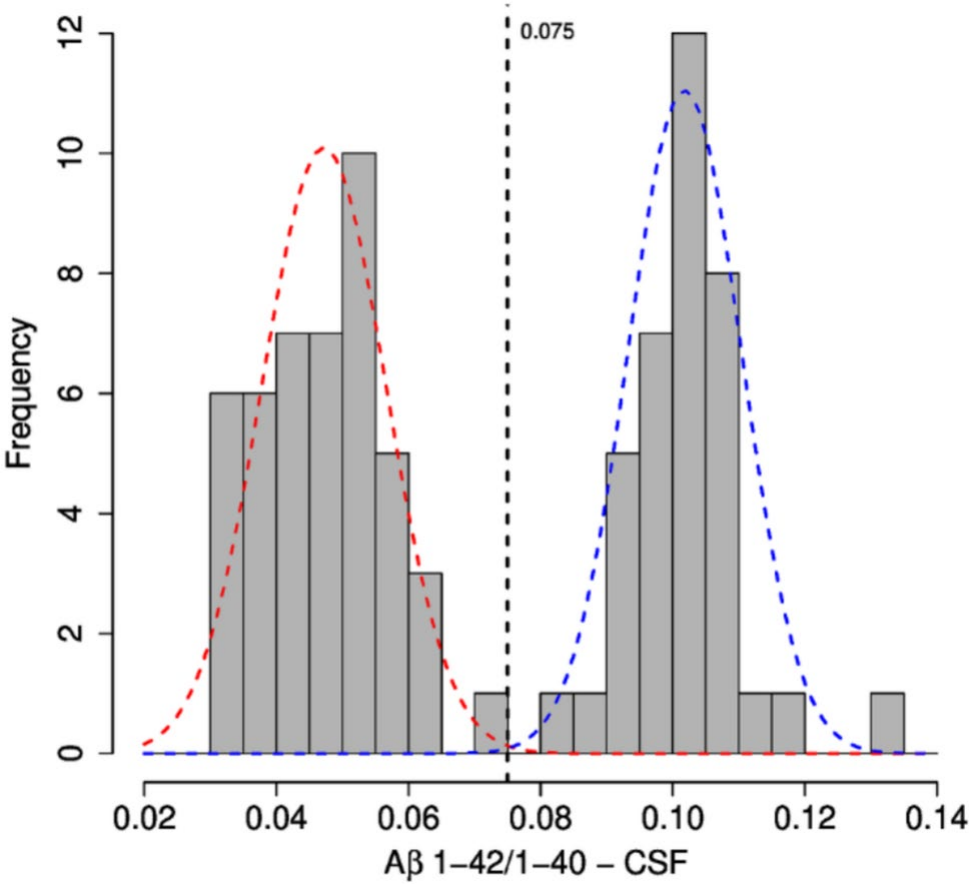
Distribution of the Aβ1–42/1–40 ratio in cerebrospinal fluid (CSF). The histogram shows the distribution of the CSF Aβ1–42/1–40 ratio determined on the LUMIPULSE System. Two normal distributions were fitted with the R package “mix‑tools” (version 1.2.0). The vertical dashed line shows the intersection of the two curves at a CSF Aβ1–42/1–40 ratio of 0.075 (threshold)

The selected study cohort comprised 82 participants for whom CSF and EDTA-blood plasma samples were available. For the statistical analyses in this study, the CSF concentrations of Aβ1–40, Aβ1–42, pTau181 and total Tau were determined with commercially available Lumipulse G CSF assays. CSF pTau217 was measured using a prototype Lumipulse G pTau217 CSF assay. Additionally, the CSF Aβ1-42/1-40 ratios were calculated, and an unbiased cutpoint for dichotomization of the study participants according to their β-amyloid status was determined. The CSF Aβ1-42/1-40 ratio showed a very clear bimodal distribution (Fig. 1), and we chose the cross-section of the two Gauss curves at a value of 0.075 as cutpoint for the dichotomization of the study cohort. The original CSF biomarker data obtained during routine from the Clinical Neurochemistry and Neurochemical Dementia Diagnostics Laboratory at the Department of Psychiatry and Psychotherapy, University of Erlangen-Nuremberg, Germany) and the clinical biomarker cutpoints were not included in the statistical analyses.

Accordingly, 45 participants were classified as Aβ-positive (CSF Aβ1–42/1–40 ≤ 0.075) and 37 as Aβ-negative (CSF Aβ1–42/1–40 > 0.075). The baseline demographic and clinical characteristics and the CSF biomarker levels assessed with Lumipulse assays are summarized in Table 1. In our sample, age and gender were statistically significantly different between the diagnostic groups. The CSF concentrations of pTau217, pTau181 and total Tau were statistically significantly increased in the Aβ-positive group.

**Table 1:** Description of the study cohort and baseline CSF biomarker data

	All	Aβ + ^a^	Aβ-^a^	p-value^b^
n (%)	82 (100%)	45 (55%)	37 (45%)	
Age [years, mean ± SD]	68.27 ± 9.59	71.49 ± 9.13	64.35 ± 8.74	< 0.001
Female (%)	38 (46%)	27 (60%)	11 (30%)	0.008^c^
APOE ε4 carriers (%)	34 (41%)	26 (58%)	8 (22%)	0.001^c^
MMSE score [mean ± SD]	25.51 ± 2.98	25.38 ± 3.23	25.67 ± 2.8	0.3877
CSF Aβ1-42/1–40 [mean ± SD]	0.07 ± 003	0.05 ± 0.01	0.10 ± 0.01	< 0.001
CSF Aβ1-42, pg/mL [mean ± SD]	864.04 ± 459.92	552.84 ± 156.63	1242.51 ± 421.51	< 0.001
CSF Aβ1-40, pg/mL [mean ± SD]	12,098.35 ± 3914.23	12,024.71 ± 3660.07	12,187.92 ± 4252.59	0.882
CSF tTau, pg/mL [mean ± SD]	557.27 ± 390.37	718.24 ± 421.48	361.49 ± 232.30	< 0.001
CSF pTau181, pg/mL [mean ± SD]	88.83 ± 70.85	123.78 ± 74.44	46.31 ± 33.71	< 0.001
CSF pTau217, pg/mL [mean ± SD]	23.93 ± 26.59	39.12 ± 27.64	5.46 ± 4.25	< 0.001

The indicated p-values for the baseline statistics were not adjusted for multiple comparisons

All of the listed CSF data were determined on the LUMIPULSE G platform

^a^The clinical sample was dichotomized according to the CSF Aβ1-42/1–40 ratio. Aβ-positive: CSF Aβ1-42/1–40 ≤ 0.075; Aβ-negative: CSF Aβ42/40 > 0.075

^b^Mann-Whitney-test p-values for the comparison of metric data between groups Aβ + and Aβ-

^c^Two-tailed Fisher test p-values for the comparison between the groups Aβ + and Aβ-

### Performance of plasma pTau217 and pTau181 for detecting low CSF Aβ1-42/1–40

Both uncorrected and age and sex adjusted levels of plasma pTau217 and pTau181 were statistically significantly increased in Aβ-positive compared to Aβ-negative subjects (Table 2). The relative differences in the medians and the standardized effect sizes (Cohen’s d) between the diagnostic groups were larger for plasma pTau217 than for plasma pTau181: the adjusted median concentration of plasma pTau217 in the Aβ-positive group was increased by 203% (3-fold increase) compared to the Aβ-negative group, while median plasma pTau181 (adjusted) was increased by 61% (1.6-fold increase). The standardized effect sizes (Cohen’s d) were calculated on log2-transformed values to account for the non-Gaussian distribution of some of the data. The resulting Cohen’s d values were 1.626 for pTau217 (adjusted for age and sex) and 1.051 for pTau181 (adjusted for age and sex) (Table 2).

**Table 2:** Direct measurement of pTau217 and pTau181 in plasma from Aβ-positive and Aβ-negative subjects

Variable	Median Aβ-	MAD^a^ Aβ-	Median Aβ +	MAD^a^ Aβ +	p-value^b^	Median Diff (%)^c^	Cohens’ d (log)^d^
pTau217 (pg/mL)	0.095	0.030	0.330	0.276	6.514E-12	247.368	2.056
pTau181 (pg/mL)	1.350	0.549	2.170	0.949	4.644E-6	60.741	1.133
pTau181/pTau217	14.225	4.358	5.922	2.296	2.29E-11	− 58.374	− 2.128
pTau217 (pg/mL) adj^e^	0.104	0.047	0.315	0.275	3.274E-9	203.281	1.626
pTau181 (pg/mL) adj^e^	1.311	0.474	2.111	0.946	9.052E-6	61.024	1.051
pTau181/pTau217 adj^e^	12.824	4.774	6.0	3.046	4.306E-10	− 53.210	1.606

^a^MAD: median absolute deviation scaled with a factor 1.4826

^b^Wilcoxon-test p-values for the comparison between the groups Aβ-positive (Aβ +) and Aβ-negative (Aβ −). After Bonferroni correction for multiple testing (6 blood-based biomarker candidates tested in total, see also Table 4) with an overall α-level of 0.05, p-values < 8.33E-3 were considered statistically significant

^c^The relative median difference between Aβ + and Aβ- groups was calculated as: ${\text{Median difference}} \left( \% \right) = 100*\frac{{{\mathrm{median}} \left( {A\beta + } \right) - {\mathrm{median}} \left( {A\beta - } \right)}}{{{\mathrm{median}}\left( {A\beta - } \right)}}$

^d^Cohen’s d were calculated after log transformation of the original data

^e^adjusted for age-and sex using a multivariate linear model on a logarithmic scale

**Fig. 2:**
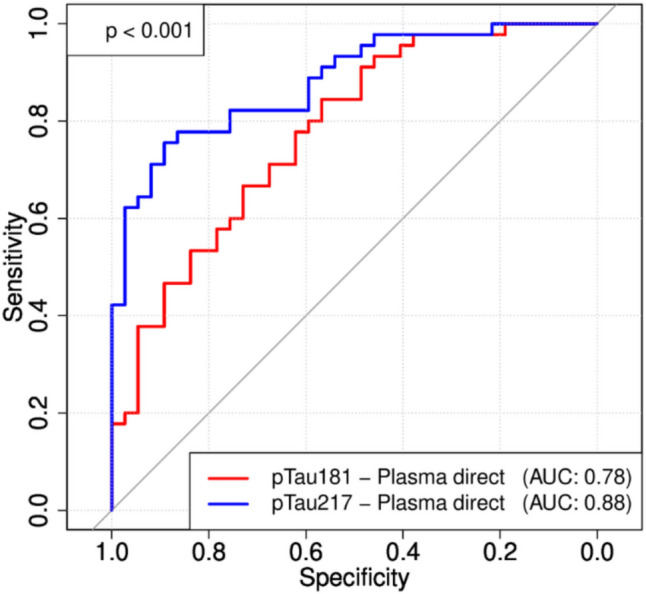
Single value ROC curves for plasma pTau217 and pTau181 (adjusted for age and sex) for the discrimination between Aβ + and Aβ- subjects according to CSF Aβ1-42/1–40

The ROC curves for the classification of the study participants into the subgroups Aβ-positive and Aβ-negative showed good performance for plasma pTau217 (adjusted for age and sex) with an AUC of 0.88 (95%CI: 0.81–0.95). The AUC for adjusted plasma pTau181 was 0.78 (95%CI 0.68–0.88), and the difference between the two reached statistical significance (p < 0.001, DeLong's test, Fig. 2). The statistics for the single value ROC analyses for the plasma biomarkers (with and without correction for age and sex) are summarized in Table 3.

**Table 3:** Statistics for single value ROC analyses evaluated at the max. Youden index^a^

Variable	AUC (95%CI)	TP	TN	FP	FN	PPV	NPV	Sensitivity	Specificity	Accuracy
pTau217	0.94 (0.90–0.99)	36	35	2	9	0.95	0.80	0.80	0.95	0.87
pTau181	0.80 (0.70–0.89)	35	27	10	10	0.78	0.73	0.78	0.73	0.75
pTau181/pTau217	0.93 (0.88–0.99)	35	36	1	10	0.97	0.78	0.78	0.97	0.88
pTau217 adj^b^	0.88 (0.81–0.95)	34	33	4	11	0.89	0.75	0.76	0.89	0.82
pTau181 adj^b^	0.78 (0.68–0.88)	38	21	16	7	0.70	0.75	0.84	0.57	0.71
pTau181/pTau217 adj^b^	0.87 ( 0.79- 0.95)	37	30	7	8	0.84	0.79	0.82	0.81	0.82

*AUC* area under the curve, *CI* confidence interval, *Tp* true positive, *Tn* true negative, *Fp* false positive, *Fn* false negative, *Ppv* positive predictive value, *Npv* negative predictive value

^a^The cut-offs were 0.19 pg/mL (0.17 pg/mL after adjustment for age and sex) for plasma pTau217, 1.54 pg/mL (1.36 pg/mL after adjustment for age and sex) for plasma pTau181 and 7.86 (9.17 after adjustment for age and sex) for pTau181/pTau217

^b^adjusted for age and sex using a multivariate linear model on a logarithmic scale

Additionally, we tentatively assessed the ratio pTau181/pTau217 as a novel biomarker candidate combining two different Tau phosphorylation sites. PTau181/pTau217 showed comparable AUC and accuracy on ROC analysis as pTau217 alone (Table 3).

### Impact of pre-analytical immunoprecipitation on the performance of plasma pTau217

To additionally assess whether the good analytical performance of the direct plasma measurements using an immunoassay could be further improved by pre-analytical sample work-up, EDTA-plasma samples were subjected to serial Aβ- and Tau-IP followed by pTau217 measurements in the resulting Tau-IP eluates. This IP-IA approach was previously shown to be able to improve the ability of a plasma pTau181 assay on the same LUMIPULSE G platform to predict brain amyloid pathology in a small sample [29].

**Fig. 3:**
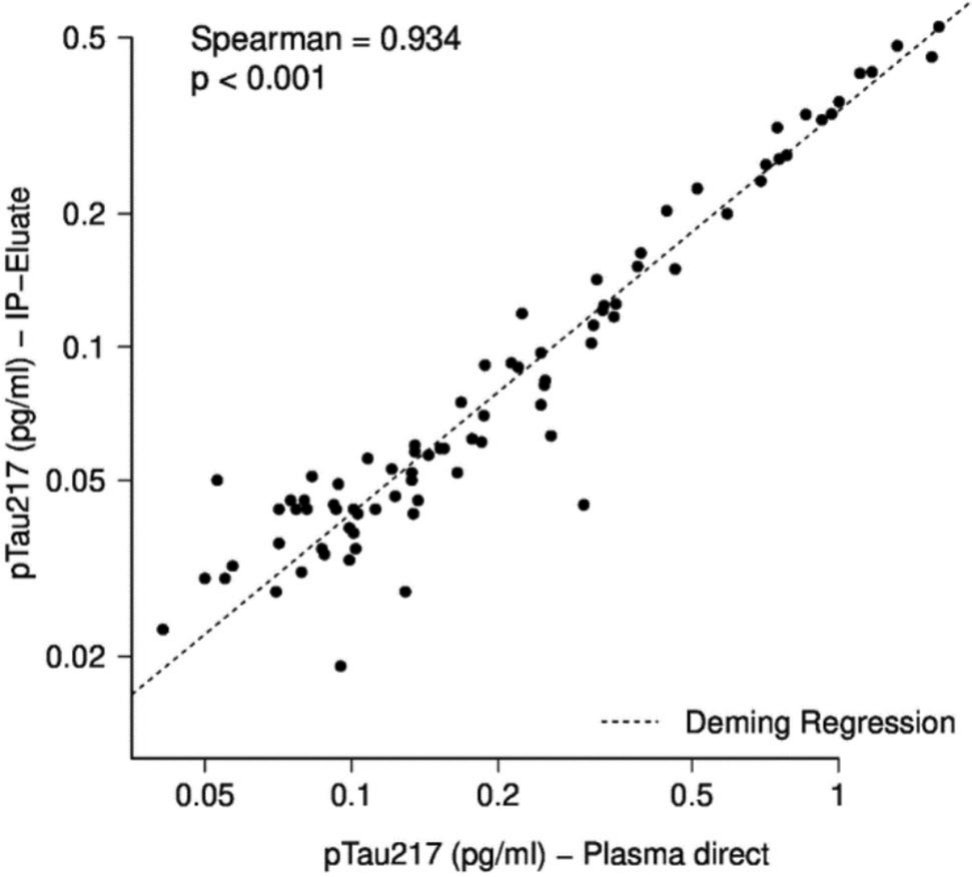
Correlation between the unadjusted concentrations of pTau217 in direct plasma and Tau-IP eluate measurements on LUMIPULSE. Diluted IP-Eluate concentrations are plotted against the corresponding direct plasma measurements. The dark grey line shows a Deming regression

Overall, the concentrations of pTau217 in the Tau-IP eluates were considerably lower than in direct plasma measurements. This can be explained by dilution introduced by the semi-automated Aβ- and Tau-IP procedures, incomplete recoveries on IP and sample preparation for the automated immunoassay [29]. However, the measured concentrations of pTau217 in plasma-IP eluates showed a very strong correlation with the corresponding direct plasma measurements (Spearman ρ = 0.934, p < 0.001, Fig. 3).

**Fig. 4:**
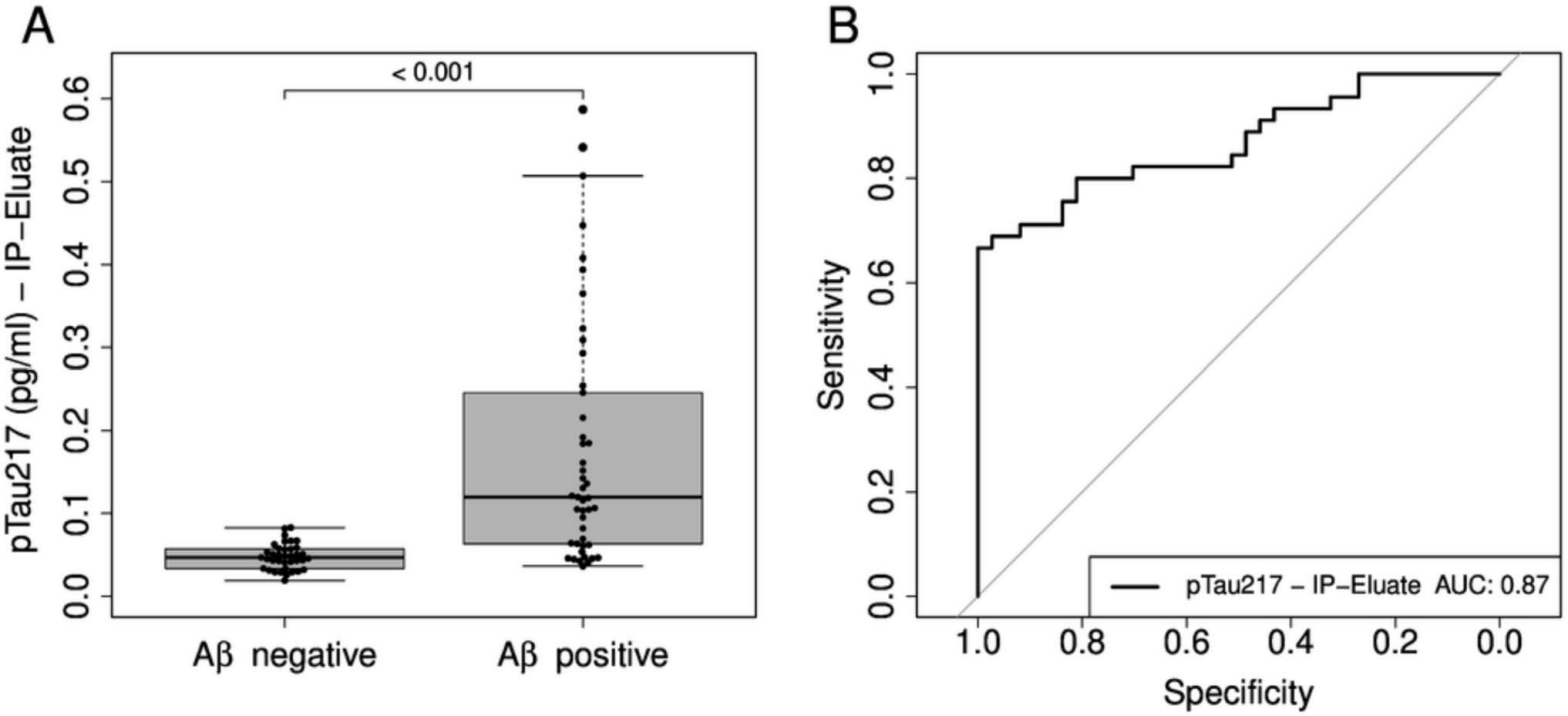
Comparison of age and sex adjusted levels of pTau217 in IP-eluates and single value ROC analysis. **A** Combined box – and scatter plots showing the age and sex adjusted concentrations of pTau217 measured on Lumipulse in IP-eluates. **B** Single value ROC curve for the detection of amyloid-positivity

The medians of the age and sex adjusted pTau217 levels in the plasma-IP eluates were increased in Aβ-positive subjects by 155% (2.5-fold increase) compared to Aβ-negative study participants (Fig. 4A and Table 4). The standardized effect size (Cohen’s d) after log transformation was 1.636, similar to that obtained for direct plasma pTau217 measurement (1.626, see Table 2).

**Table 4:** Biomarker measures in IP-eluates in Aβ-positive and Aβ-negative subjects

Variable	Median Aβ-	MAD^a^ Aβ-	Median Aβ +	MAD^a^ Aβ +	p-value^b^	Median Diff (%)^c^	Cohens’ d (log)^d^
pTau217 (pg/mL)	0.043	0.012	0.124	0.099	1.311E-12	188%	2.089
Aβ1-42/1–40	0.095	0.007	0.080	0.005	6.539E-13	-15%	-1.905
$\frac{{\mathrm{A}}\upbeta 1-40}{{\mathrm{A}}\upbeta 1-42}*{\mathrm{pTau}}217$	0.449	0.133	1.566	1.231	2.168E-18	248%	2.372
pTau217 (pg/mL) adj^e^	0.047	0.016	0.120	0.098	7.390E-10	155%	1.636
Aβ1-42/1–40 adj^e^	0.091	0.007	0.081	0.006	6.121E-09	-10%	-1.340
$\frac{{\mathrm{A}}\upbeta 1-40}{{\mathrm{A}}\upbeta 1-42}*{\mathrm{pTau}}217$ adj^e^	0.515	0.181	1.549	1.282	4.623E-12	201%	1.785

^a^MAD: median absolute deviation scaled with a factor 1.4826

^b^Wilcoxon test p-values for the comparison between the groups Aβ-positive (Aβ +) and Aβ-negative (Aβ −). After Bonferroni correction for multiple testing (6 blood-based biomarker candidates tested in total, see also Table 2) with an overall α-level of 0.05, p-value < 8.33E-3 were considered statistically significant

^c^The relative median difference between Aβ + and Aβ − groups was calculated as: ${\text{Median difference}} \left( \% \right) = 100*\frac{{{\mathrm{median}} \left( {A\beta + } \right) - {\mathrm{median}} \left( {A\beta - } \right)}}{{{\mathrm{median}}\left( {A\beta - } \right)}}$

^d^Cohen’s d were calculated after log transformation of the original data

^e^adjusted for age-and sex using a multivariate linear model on a logarithmic scale

We additionally measured the Aβ1-42/1–40 ratio in the Aβ-IP eluates from plasma on the LUMIPULSE G platform and calculated the “AT^217^-term” (Aβ1-40)/(Aβ1-42)*pTau217, which combines the Aβ1-40/1–42 ratio and pTau217 (both measured in IP-eluates) in a single biomarker value. The median Aβ1-42/1–40 ratio (after correction for age and sex) was 10% lower in the Aβ-positive study participants compared to the Aβ-negative group, and the Cohen’s d (calculated after log transformation) was -1.340. For (Aβ1-40)/(Aβ1-42)*pTau217 (adjusted for age and sex), the median in the Aβ positive group was 201% (threefold increase) higher than in the Aβ-negative group, and a statistically significantly larger (p ≤ 0.001) Cohens’ d of 1.785 than for pTau217 alone (Table 4) was found.

Single value ROC analyses revealed an AUC of 0.87 (95%CI: 0.79–0.95) for pTau217 in IP eluates (adjusted for age and sex) (Fig. 4B), similar to the AUC of 0.88 (0.81–0.95) in direct plasma measurements (p = 0.568). The adjusted Aβ1-42/1-40 ratio showed an AUC of 0.85 (95% CI: 0.77–0.94)) and the (Aβ1-40)/(Aβ1-42)*pTau217 an AUC of 0.91 (95% CI: 0.84–0.97), which was significantly superior to the AUC of pTau217 alone in IP eluates (p = 0.01, DeLong test). The statistics for the single value ROC analyses for unadjusted and adjusted biomarkers measured in IP-eluates are summarized in Table 5.

**Table 5:** Statistics for single value ROC analyses of biomarker measurements in IP-eluates evaluated at the max. Youden index

Variable	AUC (95%CI)	TP	TN	FP	FN	PPV	NPV	Sensitivity	Specificity	Accuracy
pTau217	0.96 (0.92–0.99)	37	35	2	8	0.95	0.81	0.82	0.95	0.88
Aβ1-42/1-40	0.92 (0.85–0.99)	43	32	5	2	0.9	0.94	0.96	0.86	0.91
$\frac{{\mathrm{A}}\upbeta 1-40}{{\mathrm{A}}\upbeta 1-42}*{\mathrm{pTau}}217$	0.97 (0.95–1.00)	40	26	1	5	0.98	0.88	0.89	0.97	0.93
pTau217 adj^b^	0.87 (0.79–0.95)	30	37	0	15	1	0.71	0.67	1	0.83
Aβ1-42/1-40 adj^b^	0.85 (0.77–0.94)	38	29	8	7	0.83	0.81	0.84	0.78	0.81
$\frac{{\mathrm{A}}\upbeta 1-40}{{\mathrm{A}}\upbeta 1-42}*{\mathrm{pTau}}217$ adj^b^	0.91 (0.84–0.97)	34	35	2	11	0.94	0.76	0.76	0.95	0.85

Aβ amyloid-β, AUC area under the curve, CI confidence interval, Tp true positive, Tn true negative, Fp false positive, Fn false negative, Ppv positive predictive value, Npv negative predictive value, IP immunoprecipitation

^a^The cut-offs were 0.06 pg/mL (0.09 pg/mL after adjustment for age and sex) for pTau217 in IP-eluates, 0.09 (0.09 after adjustment for age and sex) for the Aβ42/40 ratio in IP-eluates and 0.71 (0.81 after adjustment for age and sex) for (Aβ1-40)/(Aβ1-42)*pTau217

^b^adjusted for age-and sex using a multivariate linear model on a logarithmic scale

To assess whether including the ApoE4 status (absence or presence of at least one ApoE4 allele) may improve the biomarker performance, we additionally performed multivariate logistic regression ROC analyses with leave-10-out cross validation including ApoE4 status, age and sex in the model. In this statistical analysis, the numerical values of the AUCs for pTau217, Aβ1-42/1–40 and of (Aβ1-40)/(Aβ1-42)*pTau217 in IP-eluates increased marginally when the ApoE4 status was included (Table 6).

**Table 6:** Statistics of ROC analysis for biomarkers in IP-eluates by multivariate logistic regression with leave-10-out cross-validation^a^

Variable	AUC (95% CI)	Tp	Tn	Fp	Fn	Ppv	Npv	Sensitivity	Specificity	Accuracy
pTau217	0.94 (0.89–0.99)	36	35	2	9	0.95	0.8	0.8	0.95	0.87
pTau217 + ApoE4 status^b^	0.95 (0.91–0.99)	39	35	2	6	0.95	0.85	0.87	0.95	0.91
pTau217 + ApoE4 status^b^ + age + sex	0.96 (0.92–1)	40	35	2	5	0.95	0.88	0.89	0.95	0.92
Aβ1-42/1–40	0.91 (0.83–0.98)	41	32	5	4	0.89	0.89	0.91	0.86	0.89
Aβ1-42/1–40 + ApoE4 status^b^	0.92 (0.85–0.99)	44	29	8	1	0.85	0.97	0.98	0.78	0.88
Aβ1-42/1–40 + ApoE4 status^b^ + age + sex	0.91 (0.85–0.98)	37	34	3	8	0.93	0.81	0.82	0.92	0.87
$\frac{{\mathrm{A}}\upbeta 1-40}{{\mathrm{A}}\upbeta 1-42}*{\mathrm{pTau}}217$	0.96 (0.93–1.00)	41	35	2	4	0.95	0.90	0.91	0.95	0.93
$\frac{{\mathrm{A}}\upbeta 1-40}{{\mathrm{A}}\upbeta 1-42}*{\mathrm{pTau}}217+$ ApoE4 status^b^	0.97 (0.93–1.00)	41	35	2	4	0.95	0.90	091	0.95	0.93
$\frac{{\mathrm{A}}\upbeta 1-40}{{\mathrm{A}}\upbeta 1-42}*{\mathrm{pTau}}217+$ ApoE4 status^b^ + age + sex	0.97 (0.92–1)	43	35	2	2	0.96	0.95	0.96	0.95	0.95

Aβ amyloid-β, AUC area under the curve, *CI* confidence interval, *Tp* true positive, *Tn* true negative, *Fp* false positive, *Fn* false negative, *Ppv* positive predictive value, *Npv* negative predictive value, *IP* immunoprecipitation

The classification statistics were done at the maximum Youden index

^a^Multivariate logistic regression analysis was done for the Lumipulse measurements of plasma Aβ1-42/1–40 in Aβ-IP eluates and plasma pTau217 in Tau-IP eluates

^b^ApoE4 status was determined by QPCR

### Correlations

**Fig. 5:**
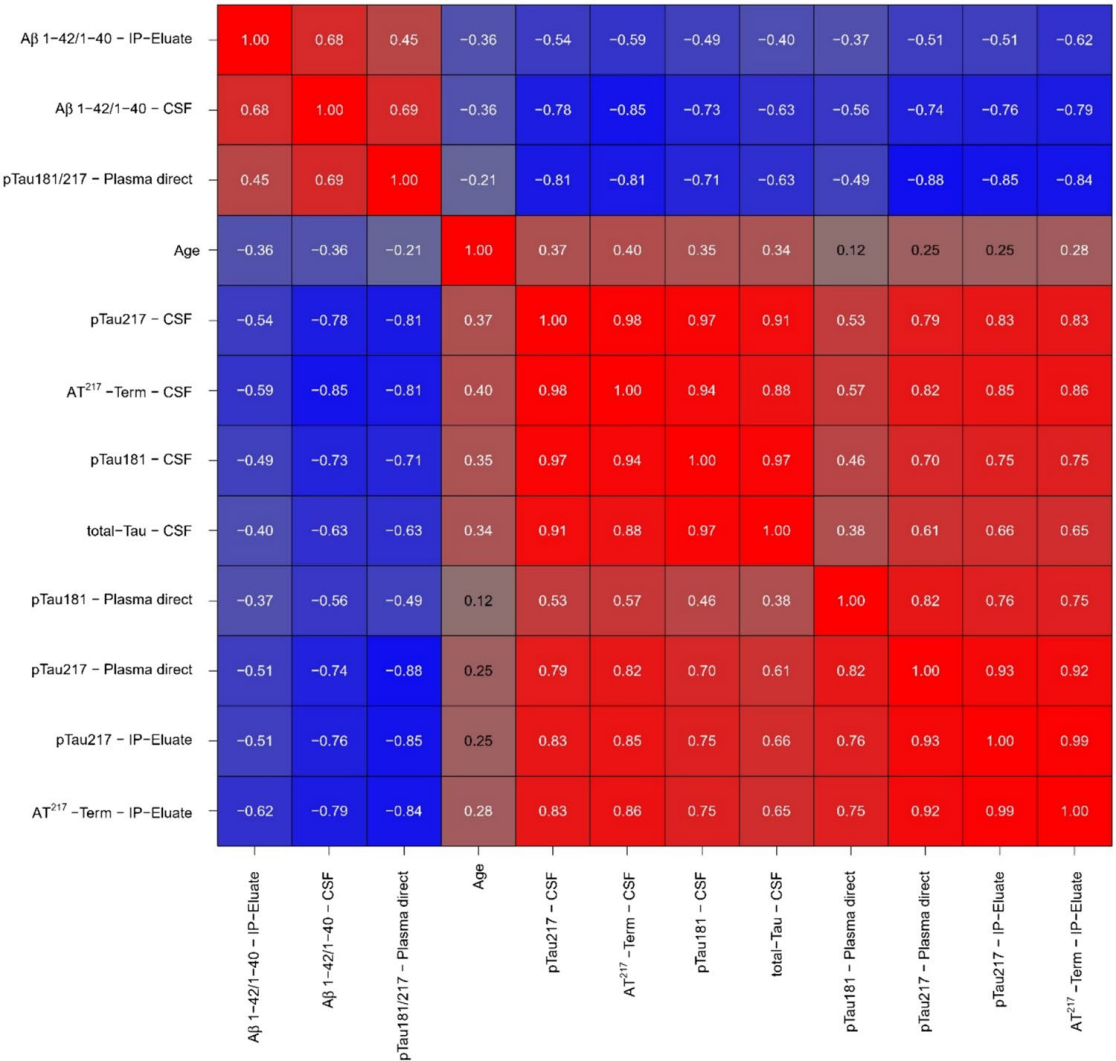
Correlation matrix (Spearman rank correlation) of all markers without adjustments for age and sex

Spearman correlations between the unadjusted markers and age were calculated and are shown in Fig. 5. For all of the listed correlations, except for age vs. plasma pTau181, uncorrected p-values < 0.05 were observed. Age showed weak correlations with the CSF and plasma biomarkers investigated. A more comprehensive correlation plot set is shown in supplementary Fig. S1. An additional age-correlation analyses is shown in supplementary Fig. S2*.*

## Discussion

Plasma pTau217 has recently emerged as a particularly promising blood-based biomarker for AD [20, 22]. Recent rapid technological developments have established sensitive and reliable immunoassays for this blood-based biomarker that are affordable and can be easily implemented in clinical routine in the upcoming years [36].

In the present exploratory study, we employed a recently developed Lumipulse assay for the measurement of plasma pTau217. The preselected pilot cohort (n = 82) included some patients in very early disease stages, and the mean MMSE scores were of 25.67 ± 2.8 and 25.38 ± 3.23 in the amyloid-negative and amyloid-positive groups, respectively. The sample was dichotomized with reference to CSF Aβ1-42/1-40 measured on Fujirebio Lumipulse using a cutpoint of 0.075, which as obtained by data driven Gaussian mixture modelling. This approach was appropriate, because in our study sample CSF Aβ1-42/1-40 showed a very clear bimodal distribution (see Fig. 1). Using the Youden Index for cutpoint determination instead and amyloid-PET for reference Keshavan et al., previously determined an identical cutpoint of 0.075 for Lumipulse CSF Aβ1-42/1-40 [37]. However, lower cutpoints of 0.062 [38] and 0.068 [39] for Lumipulse CSF Aβ 1-42/1-40 were reported in other studies. Various approaches can be applied for determining a cutpoint for dichotomized biomarkers, and the choice of a cutpoint usually involves a “trade-off” between sensitivity and specificity [40]. Consequently, the statistical approach for the determination of a cutpoint should be chosen dependent on the intended use and the population in which it is applied [40]. For example, when the biomarker test will be used for supporting a clinical diagnosis, one would likely prioritize specificity over sensitivity in order to reduce the number of false positives. In the case of the CSF Aβ42/40 ratio, choosing a lower cutpoint will reduce sensitivity but can improve specificity, while choosing a higher cutpoint is expected to increase sensitivity at the expense of lower specificity.

In our current study, the fully automated pTau217 plasma assay showed good accuracy in detecting low CSF Aβ1-42/1-40 (as a proxy of brain amyloid positivity) with a ROC-AUC of 0.88 after adjustment for age and sex. This is a large AUC value and needs to be interpreted with caution, since it may reflect the highly preselected nature of the study cohort. However, in agreement with observations from other studies employing different assays [18–20, 41], plasma pTau217 outperformed a plasma pTau181 assay for this particular context of use and in the small and preselected sample.

While the absolute levels of pTau217 in plasma were substantially lower compared to the levels of pTau181 in plasma, pTau217 showed a greater fold change in the Aβ-positive group and a larger standardized effect size (Cohen’s d). In our study, the median level of plasma pTau217 (adjusted for age and sex) was increased 3-fold in Aβ-positive individuals, which is in accordance with recent publications using the same assay on the LUMIPULSE G platform [41, 42]. Altogether, the novel plasma pTau217 assay seems to be a very promising candidate for future implementation in blood-based neurochemical routine diagnostics.

We propose the ratio pTau181/pTau217 as a novel biomarker candidate, combining the plasma measurements of both pTau217 and pTau181. We speculate that, given the different timing of abnormality onset of plasma pTau181 and pTau217 [43], this ratio could have potential diagnostic and/or prognostic relevance for severity staging and therapy monitoring across the AD continuum. In our sample, the plasma pTau181/pTau217 ratio showed a similar ROC-AUC and standardised effect size (Cohen’s d) as the single plasma pTau217 measurement. However, the dynamics of each pTau marker along the AD continuum should be further investigated.

Pre-analytical IP of Tau was previously shown to improve the diagnostic performance of plasma pTau181 for detecting low CSF Aβ42/40 [30]. The pre-analytical Tau-IP aimed to attenuate potential matrix effects by depleting plasma proteins that might interfere with the immunological measurement of plasma AD biomarkers [29, 44]. However, direct measurements of plasma pTau217 and pTau217 measurements after Tau-IP performed equally well in detecting brain amyloid pathological changes in the present study. Taken together, our observations indicated plasma pTau217 as the best single predictor of AD neuropathological changes in our sample set, with or without pre-analytical IP. To the best of our knowledge, this study investigated for the first-time plasma pTau217 with pre-analytical IP followed by IA measurement (IP-IA approach).

Several studies have shown significant improvements in diagnostic accuracy by combining two or more markers with ApoE4 genotype [45–47]. We have previously introduced the AT-term as a novel composite biomarker that combines the plasma measurements of Aβ1-40, Aβ1-42 and pTau [29]. In our present study, the ratio (Aβ1-40)/(Aβ1-42)*pTau217 in IP-eluates (adjusted for age and sex) reached a statistically significantly increased AUC of 0.91 compared to 0.87 for pTau217 alone and an increase in detection sensitivity (0.76 versus 0.67). Blood-based AD diagnostic tools should offer a high detection sensitivity since they are promising to support high throughput screening for patients at risk. Prospectively, blood-based diagnosis of AD could soon become part of the routine diagnostic work-up guiding therapeutic decisions. In this context the standardized effect size (Cohen´s d) of a biomarker is of high clinical relevance to judge the reliability of the diagnostic assay. Noteworthy, the Cohen´s d of the adjusted ratio (Aβ1-40)/(Aβ1-42)*pTau217 was significantly superior to the standardized effect size of pTau217 alone. Whether or not the ratio (Aβ1-40)/(Aβ1-42)*pTau217, preferably in direct measurements, improves the early and differential diagnosis of AD needs to be investigated in the future in larger and more diverse cohorts which should be more representative of real life clinical settings. In addition, the diagnostic performance of the (Aβ1-40)/(Aβ1-42)*pTau217 over time during real-world testing, with its inherent variability, should be investigated.

This exploratory study has limitations. The small size of the sample, the inclusion of some patients in very early disease stages, and the preselection of the participants based on the CSF Aβ42/40 ratio limit conclusions regarding generalizability of our findings. A significant selection bias cannot be excluded and the large AUC values we observed for pTau217 and the Aβ42/40 ratio may reflect the highly preselected nature or the sample. The study cohort included 60% females in the Aβ-positive and only 30% females in the Aβ-negative group and may not be representative of the population seen in a typical clinical setting. The blood collection procedure did not include a fasting protocol. However, food intake was reported to alter important blood-based AD biomarkers [48], and this and other potential confounders, for example comorbidities or medication use, need to be considered before blood-based biomarker testing can be implemented into routine clinical use. Another limitation of this study is the sole focus on the amyloid CSF status of the participants which limits conclusions regarding clinical or neuropsychological aspects. Future studies in larger and more diverse samples which are more representative of the patients seen in a typical clinical setting are needed to confirm the findings from this study.

While previous work suggested that pre-analytical sample work-up by IP might improve the performance of plasma AD biomarkers, such as Aβ1-42/1-40 ratio [28, 44] and pTau181 [29], to predict brain amyloid pathology, the same was not observed for pTau217 in the current study. The sequential IP of Aβ and Tau from the same blood plasma aliquot offers the possibility of analysing several biomarkers extracted from a single initial sample aliquot. With larger volumes of plasma samples available the concentration of biomarkers can be increased by the IP procedure and interfering matrix effects can be reduced. However, future studies have to demonstrate if a potential increase in diagnostic accuracy and reliability will justify the additional costs, time-consuming sample treatment and potential risk of additional technical errors which may limit the potential implementation in clinical routine introduced by the additional IP protocol prior immunological biomarker measurements.

In summary, our study suggests that the fully automated Lumipulse G pTau 217 Plasma and Lumipulse G pTau181 Plasma assays are highly effective in detecting AD pathophysiology. It appears that pTau217 is one of the most promising plasma biomarkers for future implementation in routine clinical practice. Our results are in line with previous studies using the LUMIPULSE G System [41, 42]. The availability of plasma biomarkers on accessible and efficient fully automated platforms will be essential for pre-screening and monitoring the efficacy of novel disease-modifying treatments.

## Supplementary Information

Below is the link to the electronic supplementary material.

Supplementary file1 (DOCX 845 KB)

Supplementary file2 (PDF 317 KB)

Supplementary file3 (PDF 28 KB)

## Data Availability

The datasets used and/or analysed in the present study are available from the corresponding authors on reasonable request.
