# Supplementary material for: Evaluation of a fully automated assay for the measurement of plasma pTau217 and a composite score integrating the ratio Aβ1-42/1-40 as biomarkers of Alzheimer’s disease
Source: Eur Arch Psychiatry Clin Neurosci. 2025 Sep 27;276(5):2251–64. doi: 10.1007/s00406-025-02123-8 (PMC13481745; doi:10.1007/s00406-025-02123-8)
Supplement: Supplementary file 1 [file 406_2025_2123_MOESM2_ESM.pdf]

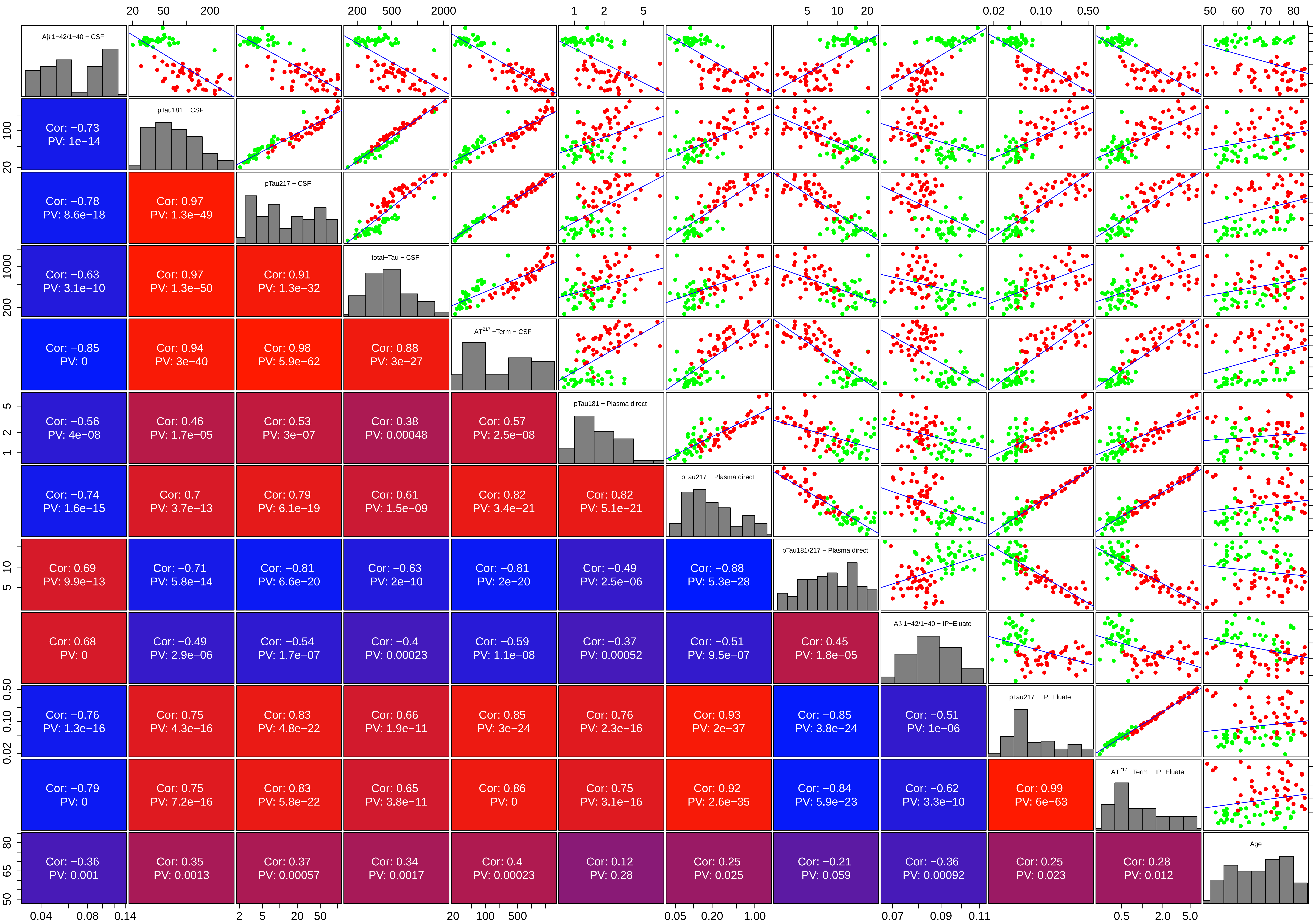

Aβ 1−42/1−40 − CSF
pTau181 − CSF
pTau217 − CSF
total−Tau − CSF
AT217 −Term − CSF
pTau181 − Plasma direct
pTau217 − Plasma direct
pTau181/217 − Plasma direct
Aβ 1−42/1−40 − IP−Eluate
pTau217 − IP−Eluate
AT217 −Term − IP−Eluate
Age
Cor: −0.73 PV: 1e−14
Cor: −0.78 PV: 8.6e−18
Cor: 0.97 PV: 1.3e−49
Cor: −0.63 PV: 3.1e−10
Cor: 0.97 PV: 1.3e−50
Cor: 0.91 PV: 1.3e−32
Cor: −0.85 PV: 0
Cor: 0.94 PV: 3e−40
Cor: 0.98 PV: 5.9e−62
Cor: 0.88 PV: 3e−27
Cor: −0.56 PV: 4e−08
Cor: 0.46 PV: 1.7e−05
Cor: 0.53 PV: 3e−07
Cor: 0.38 PV: 0.00048
Cor: 0.57 PV: 2.5e−08
Cor: −0.74 PV: 1.6e−15
Cor: 0.7 PV: 3.7e−13
Cor: 0.79 PV: 6.1e−19
Cor: 0.61 PV: 1.5e−09
Cor: 0.82 PV: 3.4e−21
Cor: 0.82 PV: 5.1e−21
Cor: 0.69 PV: 9.9e−13
Cor: −0.71 PV: 5.8e−14
Cor: −0.81 PV: 6.6e−20
Cor: −0.63 PV: 2e−10
Cor: −0.81 PV: 2e−20
Cor: −0.49 PV: 2.5e−06
Cor: −0.88 PV: 5.3e−28
Cor: 0.68 PV: 0
Cor: −0.49 PV: 2.9e−06
Cor: −0.54 PV: 1.7e−07
Cor: −0.4 PV: 0.00023
Cor: −0.59 PV: 1.1e−08
Cor: −0.37 PV: 0.00052
Cor: −0.51 PV: 9.5e−07
Cor: 0.45 PV: 1.8e−05
Cor: −0.76 PV: 1.3e−16
Cor: 0.75 PV: 4.3e−16
Cor: 0.83 PV: 4.8e−22
Cor: 0.66 PV: 1.9e−11
Cor: 0.85 PV: 3e−24
Cor: 0.76 PV: 2.3e−16
Cor: 0.93 PV: 2e−37
Cor: −0.85 PV: 3.8e−24
Cor: −0.51 PV: 1e−06
Cor: −0.79 PV: 0
Cor: 0.75 PV: 7.2e−16
Cor: 0.83 PV: 5.8e−22
Cor: 0.65 PV: 3.8e−11
Cor: 0.86 PV: 0
Cor: 0.75 PV: 3.1e−16
Cor: 0.92 PV: 2.6e−35
Cor: −0.84 PV: 5.9e−23
Cor: −0.62 PV: 3.3e−10
Cor: 0.99 PV: 6e−63
Cor: −0.36 PV: 0.001
Cor: 0.35 PV: 0.0013
Cor: 0.37 PV: 0.00057
Cor: 0.34 PV: 0.0017
Cor: 0.4 PV: 0.00023
Cor: 0.12 PV: 0.28
Cor: 0.25 PV: 0.025
Cor: −0.21 PV: 0.059
Cor: −0.36 PV: 0.00092
Cor: 0.25 PV: 0.023
Cor: 0.28 PV: 0.012
