# Supplementary material for: Evaluation of a fully automated assay for the measurement of plasma pTau217 and a composite score integrating the ratio Aβ1-42/1-40 as biomarkers of Alzheimer’s disease
Source: Eur Arch Psychiatry Clin Neurosci. 2025 Sep 27;276(5):2251–64. doi: 10.1007/s00406-025-02123-8 (PMC13481745; doi:10.1007/s00406-025-02123-8)
Supplement: Supplementary file 2 [file 406_2025_2123_MOESM3_ESM.pdf]

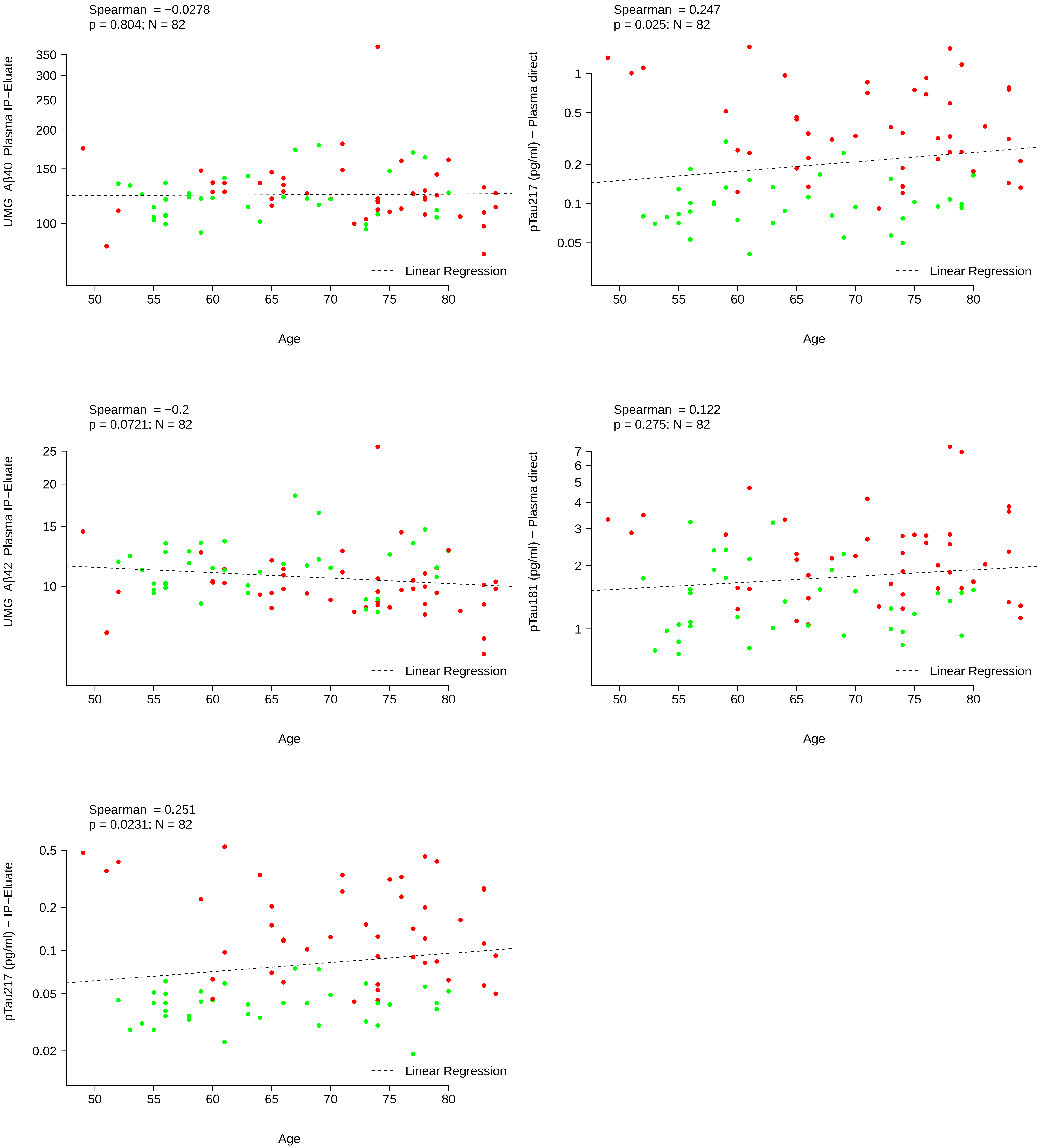

Spearman = −0.0278
p = 0.804; N = 82
UMG Aβ40 Plasma IP−Eluate
350
300
250
200
150
100
Linear Regression
50 55 60 65 70 75 80
Age
Spearman = 0.247
p = 0.025; N = 82
pTau217 (pg/ml) − Plasma direct
1
0.5
0.2
0.1
0.05
Linear Regression
50 55 60 65 70 75 80
Age
Spearman = −0.2
p = 0.0721; N = 82
UMG Aβ42 Plasma IP−Eluate
25
20
15
10
Linear Regression
50 55 60 65 70 75 80
Age
Spearman = 0.122
p = 0.275; N = 82
pTau181 (pg/ml) − Plasma direct
7
6
5
4
3
2
1
Linear Regression
50 55 60 65 70 75 80
Age
Spearman = 0.251
p = 0.0231; N = 82
pTau217 (pg/ml) − IP−Eluate
0.5
0.2
0.1
0.05
0.02
Linear Regression
50 55 60 65 70 75 80
Age
