# Supplementary material for: Evaluation of a fully automated assay for the measurement of plasma pTau217 and a composite score integrating the ratio Aβ1-42/1-40 as biomarkers of Alzheimer’s disease
Source: Eur Arch Psychiatry Clin Neurosci. 2025 Sep 27;276(5):2251–64. doi: 10.1007/s00406-025-02123-8 (PMC13481745; doi:10.1007/s00406-025-02123-8)
Supplement: Supplementary file 3 [file 406_2025_2123_MOESM1_ESM.docx]

**Supplementary material**

**Evaluation of a fully automated assay for the measurement of plasma pTau217 and a composite score integrating the ratio Aβ1-42/1-40 as biomarkers of Alzheimer’s disease**

Barbara Morgado^1^*, Hans-Wolfgang Klafki^1^*, Chris Bauer^2^, Annik Steiert^1^, Katharina Waniek^3^, Ingolf Lachmann^3^, Dirk Osterloh^3^, Hermann Esselmann^1^, Niels Hansen^1^, Johannes Schuchhardt^2^, Jens Wiltfang^1, 4, 5^

^1^ Department of Psychiatry and Psychotherapy, University Medical Center Goettingen (UMG), Georg-August University, Von-Siebold Strasse 5, 37075 Goettingen, Germany

^2^ MicroDiscovery GmbH, Marienburger Strasse 1, 10405 Berlin, Germany

^3^ Roboscreen GmbH, Hohmannstrasse 7, 04129 Leipzig, Germany

^4^ German Center for Neurodegenerative Diseases (DZNE), 37075 Goettingen, Germany

^5^ Neurosciences and Signaling Group, Institute of Biomedicine (iBiMED), Department of Medical Sciences, University of Aveiro, 3810-193 Aveiro, Portugal

* Barbara Morgado and Hans-Wolfgang Klafki contributed equally to this work

**Corresponding author**

Barbara Morgado (barbara.marcosmorgado@med.uni-goettingen.de)

**Content:**

**Supplementary Figure S1**. Pairwise correlation analysis of biomarker measures in CSF, plasma and eluates obtained after immunoprecipitation from EDTA-plasma.

**Supplementary Figure S2.** Analysis of Spearman correlations between age and measurements in plasma or IP-eluates.

**Supplementary Figure S1. Pairwise correlation analysis of biomarker measures in CSF, plasma and eluates obtained after immunoprecipitation from EDTA-plasma.**

**Supplementary Figure S1.** Pairwise correlation analysis of biomarker measures in CSF, plasma and eluates obtained after immunoprecipitation from EDTA-plasma. The heatmap shows Spearman rank correlation coefficients and p-values (PV). Data points in the scatter plots are coloured by diagnostic group (red = Aβ+, green = Aβ-). All variables except for the age are shown on log axes. The blue line corresponds to the linear regression.

**Supplementary Figure S2.** Analysis of Spearman correlations between age and measurements in plasma or IP-eluates.

**Supplementary Figure 2**: Analysis of Spearman correlations between age and measurements in plasma or IP-eluates. Red dots: Aβ-positive study participants, green dots: Aβ-negative study participants. The indicated p-values were not adjusted for multiple testing.
